# Higher levels of *Pseudomonas aeruginosa* LasB elastase expression are associated with early-stage infection in cystic fibrosis patients

**DOI:** 10.1038/s41598-023-41333-9

**Published:** 2023-08-30

**Authors:** Agustina Llanos, Pauline Achard, Justine Bousquet, Clarisse Lozano, Magdalena Zalacain, Carole Sable, Hélène Revillet, Marlène Murris, Marie Mittaine, Marc Lemonnier, Martin Everett

**Affiliations:** 1 Antabio SAS, Biostep, 436, rue Pierre et Marie Curie, 31760 Labège, France; 2 Service de Bactériologie-Hygiène, CHU de Toulouse, Toulouse, France; 3 IRSD, INSERM, Université de Toulouse, INRAE, ENVT, UPS, Toulouse, France; 4 Adult Cystic Fibrosis Centre, Pulmonology Unit, Hôpital Larrey, CHU de Toulouse, Toulouse, France; 5 Hôpitaux de Toulouse, Toulouse, France

**Keywords:** Cystic fibrosis, Translational research, Bacterial pathogenesis

## Abstract

*Pseudomonas aeruginosa* is a common pathogen in cystic fibrosis (CF) patients and a major contributor to progressive lung damage. *P. aeruginosa* elastase (LasB), a key virulence factor, has been identified as a potential target for anti-virulence therapy. Here, we sought to differentiate the *P. aeruginosa* isolates from early versus established stages of infection in CF patients and to determine if LasB was associated with either stage. The *lasB* gene was amplified from 255 *P. aeruginosa* clinical isolates from 70 CF patients from the Toulouse region (France). Nine LasB variants were identified and 69% of the isolates produced detectable levels of LasB activity. Hierarchical clustering using experimental and clinical data distinguished two classes of isolates, designated as ‘Early’ and ‘Established’ infection. Multivariate analysis revealed that the isolates from the Early infection class show higher LasB activity, fast growth, tobramycin susceptibility, non-mucoid, pigmented colonies and wild-type *lasR* genotype. These traits were associated with younger patients with polymicrobial infections and high pFEV_1_. Our findings show a correlation between elevated LasB activity in *P. aeruginosa* isolates and early-stage infection in CF patients. Hence, it is this patient group, prior to the onset of chronic disease, that may benefit most from novel therapies targeting LasB.

## Introduction

Cystic fibrosis (CF) is a genetic disease caused by a mutation leading to the complete or partial loss-of-function of the CF transmembrane conductance regulator (CFTR), a protein responsible for ion transport across apical membranes of epithelial cells. CF is a multisystemic disease affecting lung, pancreas and digestive tract, male reproductive system and other glandular organs^1^. The CF lungs are prone to infections, and *Pseudomonas aeruginosa* is one of the most common bacteria to colonize this niche^2^. *P. aeruginosa* is a gram-negative opportunistic pathogen, ubiquitous in nature, whose versatile genome and significant functional adaptability allow it to thrive in diverse environments^3^. *P. aeruginosa* adaptation to the harsh environment and the selective pressure of the CF lung leads to the establishment of chronic infection, resistance to antibiotics and loss of virulence factors such as motility, type III secretion, exotoxins, and proteases^4–6^. In the CF lung, the establishment of a *P. aeruginosa* chronic infection has been correlated to dysbiosis and the development of a disease-promoting microbiome^7^. *P. aeruginosa* chronic infections have also been described in other respiratory diseases, such as non-CF bronchiectasis (NCFB) and chronic obstructive pulmonary disease (COPD)^8,9^.

The success of *P. aeruginosa* colonization resides in its capacity to produce a large variety of virulence factors, including toxins and proteases^10,11^, under the control of a quorum sensing (QS) regulatory system^12,13^. The QS system is composed of four known pathways, namely the LasR/LasI, the RhlR/RhlI, the PqsR controlled quinolones system and the IQS system. The regulators are organized in a hierarchy, with LasR at the top^14^. The LasB elastase, the most abundant protein in the secretome, is a key virulence factor expressed under the control of the positive regulator LasR^15,16^. Indeed, the expression of LasB can be dramatically reduced in Δ*lasR* mutants^17^ or by the addition of quorum sensing inhibitors, such as trans-cinnamaldehyde, to the culture medium^18^. This extracellular protease has numerous bacterial and host substrates and provokes tissue damage and inflammation in the host^19,20^. Recently, a study showed that LasB also promotes eosinophil infiltration and mucin production^21^. Furthermore, the presence of LasB has been shown to be important in the establishment of chronic infection in mice^22^.

LasB has been identified as a potential target for anti-virulence therapy, as an alternative or adjunct to antibiotics^19^. To better understand how such a therapy could be targeted to treat CF patients, we sought to differentiate *P. aeruginosa* isolates from early versus established (chronic) stages of CF infection and determine if LasB expression/activity was associated with either stage. In this study, we investigated the presence of *lasB* and *lasR* genes, the prevalence of different LasB variants and the production of active LasB in 255 *P. aeruginosa* sputum isolates from 70 CF patients. We applied a multivariate analysis, using both clinical and experimental data, to identify the variables which discriminate between isolates from early and established infections and determine whether either group is more strongly associated with higher levels of LasB expression.

## Results

### Patient population and *P. aeruginosa* isolates

The 255 *P. aeruginosa* strains used in this study were isolated from sputum samples collected from 70 CF patients between January 2015 and June 2020 (1–14 isolates per patient) (Table 1 and Supplementary Fig. S1). Both paediatric and adult patients were included and the median age (at the time of the first sample) was 12 years (range 1–47). pFEV_1_ was recorded at the time of sampling, except for younger patients (< 6 years). CFTR genotypes were available for all but seven patients; 36 (51%) carried mutations leading to abnormal processing and trafficking (Class II) in both alleles of the CFTR locus^23,24^ of which 32 were homozygous for the F508 deletion. The infection status of patients was classified according to the ‘Leeds criteria’, as Chronic (*P. aeruginosa* isolated from > 50% of samples in the last 12 months), Intermittent (≤ 50% of samples in the last 12 months) or New (*P. aeruginosa* isolated for the first time)^25,26^. Where multiple *P. aeruginosa* isolates were obtained from the same patient over the course of the study, the classification evolved accordingly, hence each isolate was associated with the infection status of the patient at the time of sampling. According to these criteria, 26% of the patients had a Chronic *P. aeruginosa* infection diagnosed either before or during the period of the study.

**Table 1:** Characteristics of the cohort of patients included in this study.

Number of patients	70
Number of isolates	255
Number of isolates per patient, median (range)	2 (1–14)
Age*, median (range)	12 (1–47)
CFTR class (number of patients = 70)	
II/II (homozygous F508del)	36 (32)
I/Unclassified	8
II/Unclassified	7
II/I	4
Others	8
Unknown	7

*Age at the time of the first sample included in the study.

For 137 isolates, the data was available on mucoid phenotype, which was recorded immediately after their isolation from the sputum samples. Information on colony size and pigmentation was subsequently obtained for a separate, but overlapping, subset of 98 *P. aeruginosa* isolates which had been selected for the multivariate analysis. The proportion of isolates displaying distinct phenotypic traits within each Leeds category was compared with the expected proportion if these traits were randomly distributed in the population using a Chi2 test (Supplementary Table S1 and Supplementary Fig. S2). Among the Chronic isolates, 47% had very small punctiform colonies, 47% were mucoid and 31% had pigmented colonies. The first two traits were higher than the expected proportion (adjusted *p*-values: 1.33 × 10^−3^ and 1.69 × 10^−5^, respectively), whereas the last one (pigmentation) was lower than expected (adjusted *p*-value: 0.02). In the Intermittent group, 16% of isolates had very small colonies, 68% had pigmentation (not significant) and none of them were mucoid (adjusted *p*-value: 1.89 × 10^−3^). Finally, in the New group, 5% of isolates had very small colonies and 7% were mucoid, both of which were lower than the expected proportion (adjusted *p*-values: 1.98 × 10^−2^ and 0.028, respectively), and 66% were pigmented (not significant).

### Identification of the LasB and LasR variants in *P. aeruginosa* isolates

The *lasB* gene was successfully amplified and sequenced from all 255 *P. aeruginosa* isolates. Nine LasB protein variants were identified, of which three together accounted for 94.5% of the isolates (Table 2), namely LasB-1 (identical to the PAO1 wild-type sequence), LasB-2 (single S241G amino-acid change) and LasB-3 (five amino-acid changes: Q102R, S241G, D244N, K282N, R471S). Six other variants with one, two or five amino-acid modifications, were detected in one to five isolates. Analysis of the Pseudomonas.com genome database^27^ (7960 genomes with 99.1% including a full length *lasB* locus at the time of writing) revealed the same three major variants, namely LasB-1 (47.4%), LasB-2 (17.3%) and LasB-3 (22.6%). Five of the six minor variants found in this study were also represented in the Pseudomonas.com database, which contained an additional 140 variants. As already reported elsewhere^28^, seven of the eight LasB variants, including the three major variants, had similar LasB specific activities with regard to hydrolysis of the Abz substrate within two-fold of each other, with the only exception being LasB-9 that showed lower activity (Table 2).

**Table 2:** Prevalence of the different LasB variants in the isolate collection from this study and comparison to the LasB variants from the genomes available in the Pseudomonas.com database. The *lasB* gene was amplified and sequenced from all the isolates included in this study. The LasB sequence from the *P. aeruginosa* PAO1 strain was considered the reference sequence (WT; LasB-1).

Variant ID	AA substitutions	This study (n = 255)		Pseudomonas.com database (n = 7890)*		LasB specific activity†
		Number of strains	Prevalence in the population (%)	Number of strains	Prevalence in the population (%)	
LasB-1 (WT)	WT	120	47.1	3738	47.4	6.5
LasB-2	S241G	70	27.4	1363	17.3	6.3
LasB-3	Q102R, S241G, D244N, K282N, R471S	51	20.0	1787	22.6	6.3
LasB-4	T65I	3	1.2	29	0.4	5.4
LasB-5	Q71L	3	1.2	1	0.01	3.3
LasB-6	M325V	1	0.4	58	0.7	ND
LasB-7	S460T	5	1.9	18	0.2	6.9
LasB-8	S241G, A497S	1	0.4	71	0.9	ND
LasB-9	N68S, T120I, A122T, S241G, S436L	1	0.4	0	0	1.6
Other variants		–	–	825	10.5	–

*Number of genomes containing a full-length *lasB* gene at the time of writing.

^†^Mean specific activity (RFU/min/mg protein × 10^8^) measured using the Abz-AGLA-Nba substrate.

Amplification and sequencing of the *lasR* locus was successfully achieved for all but one isolate. Of these, the predicted proteins were identical to LasR from PAO1 (WT) in 162 (63.8%) isolates, whereas 36 (14.2%) carried mutations leading to a truncated protein and 56 (22.0%) contained one or more amino-acid mutations or small deletions (2 or 5 amino-acids) (Supplementary Table S2). For comparison, in the Pseudomonas.com genome database, the *lasR* locus was absent in 1081 genomes (13.6%). Among those genomes containing *lasR*, 57.3% were WT, 30.8% encoded a LasR variant and 11.9% a truncated LasR. Although the percentage of WT LasR is high, this locus presents considerably greater variability than that observed for *lasB*.

### LasB activity in *P. aeruginosa* isolates from CF patients

The activity of LasB was determined by following the hydrolysis of the fluorescent substrate Abz in the supernatants of *P. aeruginosa* cultures. Of the 255 isolates, 176 (69%) showed LasB activity, whereas 79 (31%) were below the positive threshold for detection. The 79 negative samples were further analysed by Western blot to test for LasB protein production (examples presented in Supplementary Fig. S3). Of these, 29 showed a 33 kDa band corresponding to the LasB protein, whereas 50 showed no LasB protein. Of this latter group, 43 (86%) contained a mutation in *lasR*, with 27 (54%) of these predicted to lead to an inactive LasR protein.

**Figure 1:**
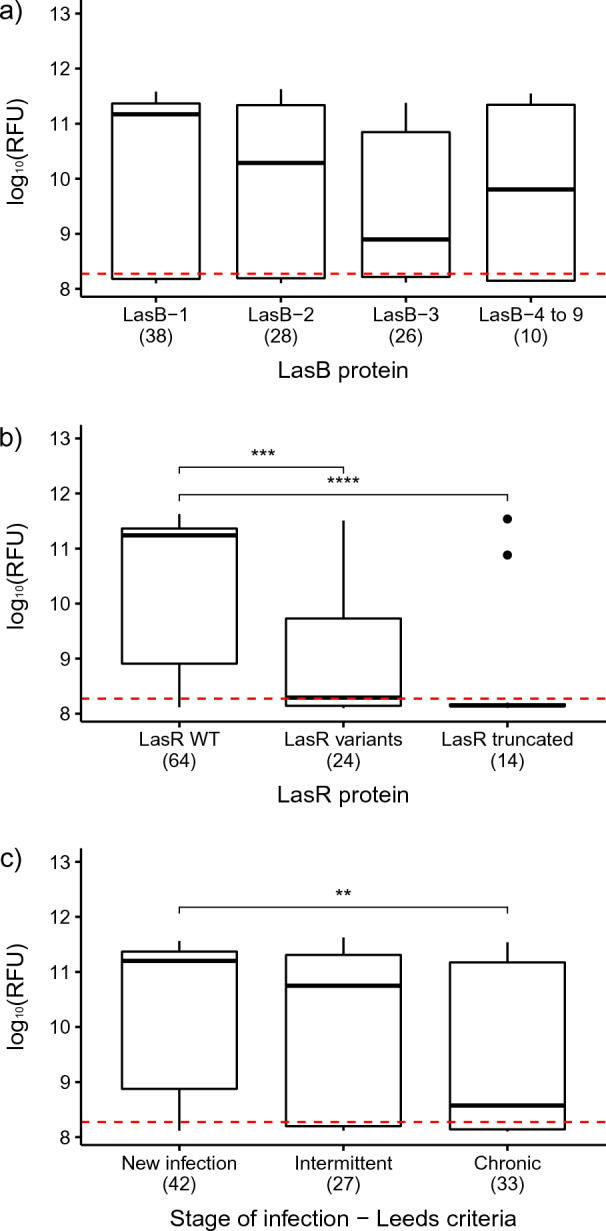
Distribution of the LasB activity, as measured by the Abz hydrolysis, in *P. aeruginosa* isolates depending on (**a**) LasB variant, (**b**) LasR variant and (**c**) Leeds’ stages of infection. The hydrolysis of the Abz substrate in the Y axis is expressed as the log_10_(RFU). The lower and upper boundaries of the box represent the 25th and the 75th percentile, respectively. The black line within the box represents the median. The outliers are represented by the circles. The red dashed line indicates the positive threshold of the assay. Significant differences are indicated by the stars on top of the boxplots. *p*-value: **< 0.01; ***< 0.001; ****< 0.0001.

To minimise bias due to the occurrence of multiple, contemporaneous isolates from the same patient and thus facilitate a more meaningful statistical analysis, the database was refined following the workflow presented in the Supplementary Fig. S4. Briefly, where there was more than one isolate from each stage of infection from any one patient, representatives were selected which were phenotypically or genotypically distinct based on their LasB activity and *lasB*/*lasR* genotypes. This resulted in a dataset of 102 ‘unique’ isolates which was used to investigate the correlation between LasB activity, as measured by Abz hydrolysis, and other variables. Within this set, 70 isolates (68.6%) demonstrated a detectable level of Abz hydrolysis, i.e., LasB positive, and 32 (31.4%) were negative. The median age of the LasB positive group was 12 years and the median pFEV_1_ was 85, whereas for the LasB negative group, the median age was 22 years and the median pFEV_1_ was 71 (Table 3). There were no significant differences in LasB activity between LasB variants (median range: 7.76 × 10^8^–1.48 × 10^11^) (Fig. 1a; statistical tests and *p*-values are presented in Supplementary Table S3). In contrast, isolates with WT LasR showed significantly higher elastase activity (median: 1.74 × 10^11^) than those with a variant (median: 1.95 × 10^8^) (amino-acid substitutions or small deletions) or truncated (median: 1.41 × 10^8^) form of LasR (Fig. 1b). Finally, those isolates from patients designated as having Chronic infections showed significantly lower LasB activity (median: 3.72 × 10^8^) than those from New infections (median: 1.58 × 10^11^) (Fig. 1c).

**Table 3:** Characteristics of the isolates in the refined dataset, comparing the positive versus negative isolates for the Abz hydrolysis.

	Total	Positive LasB activity	Negative LasB activity
**Number of isolates**	102	70	32
**Patient characteristics**, median (range)			
Age	13 (1–46)	12 (1–37)	22 (2–46)
pFEV_1_	83 (30–125)	85 (31–125)	71 (30–111)
**Stage of infection (Leeds criteria)**, number of isolates			
New isolates	42	34	8
Intermittent isolates	27	19	8
Chronic isolates	33	17	16
**LasB type**, number of isolates			
LasB-1 (WT)	38	26	12
LasB-2	28	20	8
LasB-3	26	18	8
LasB-4 to LasB-9	10	6	4
**LasR type**, number of isolates			
LasR WT	64	56	8
LasR variants	24	13	12
LasR truncated	14	2	12

### Classification of the *P. aeruginosa* isolates and identification of discriminant variables

Initially, a supervised multivariate analysis (sPLS-DA) was performed on the 102 isolates from the refined dataset to identify, (i) the variables that discriminate between the three Leeds stages of infection (New, Intermittent and Chronic) and (ii) which variables correlate with the presence of LasB activity. The variables used for this analysis included patient clinical data (age, pFEV_1_, time since first infection, infection within the last 3 months prior to the date of collection of each isolate, CFTR class), phenotypic characteristics of the isolate (Abz hydrolysis, bacterial growth, susceptibility to tobramycin and aztreonam, mucoidy and colony morphology) and genotypic data (LasB and LasR variants) (Supplementary Datafile). The model showed good separation between the New and Chronic isolates, however, the Intermittent group was loosely defined and overlapped both Chronic and New groups (Supplementary Fig. S5). Therefore, this model did not discriminate clearly between all three groups and showed a high cross-validation error (32.1%).

**Figure 2:**
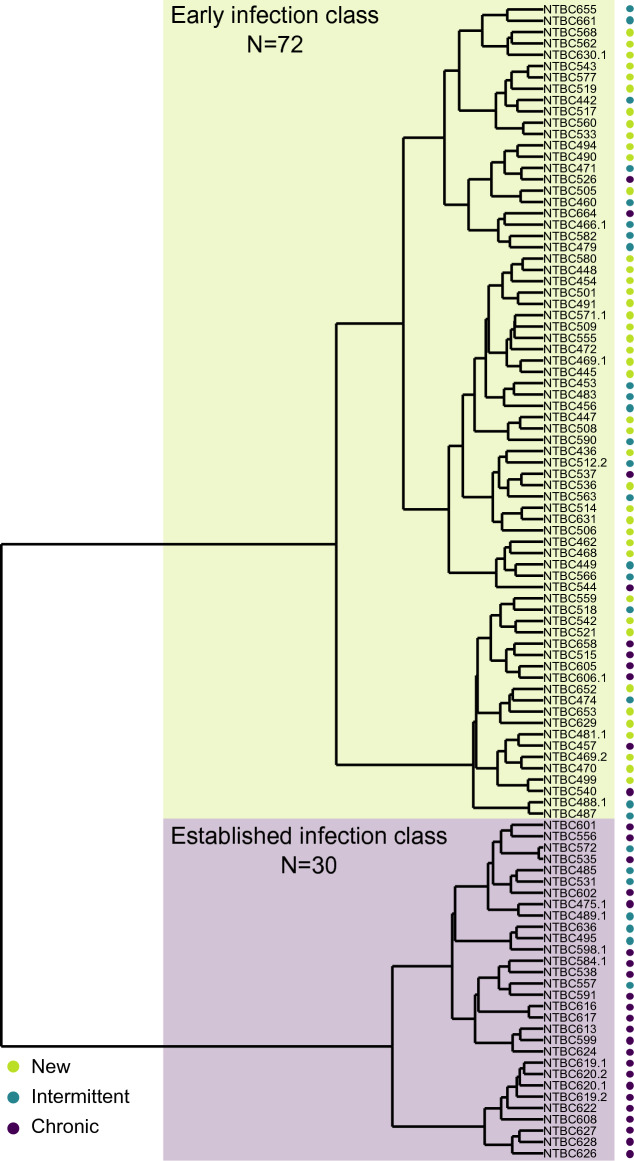
Hierarchical clustering of the refined dataset isolates into Early infection class (green) and Established infection (violet). The dots on the right indicate to which Leeds’ stage of infection each isolate belongs.

To generate an improved model that could provide information about the LasB activity and correlated variables, the same 102 isolates from the refined dataset were first classified using a hierarchical clustering method. In this analysis, the Leeds stage of infection variable was excluded, but all the other available variables were retained. This new clustering revealed two distinct classes (Fig. 2). Seventy-two isolates clustered into the first class, of which 58.3% had previously been designated as New, 27.8% Intermittent and only 13.9% as Chronic, according to the Leeds criteria. The second class comprised 30 isolates, of which 76.7% were Chronic and 23.3% were Intermittent. No New isolates were present in this class. Moreover, in the first class, a higher abundance of WT LasR (68%) and a higher level of Abz hydrolysis (median: 1.5 × 10^11^) was observed compared to the second class (50% WT LasR, Abz hydrolysis median: 1.56 × 10^8^, Supplementary Table S4). We designated the first and second class as ‘Early infection’ and ‘Established infection’, respectively.

**Figure 3:**
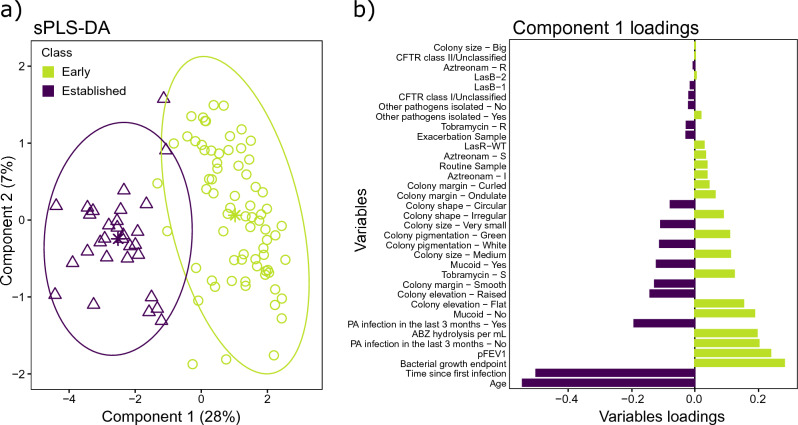
sPLS-DA to discriminate between the Early and Established infection classes and identify the discriminant variables. (**a**) Individual plots of the two infection groups. (**b**) Component 1 contributing variables’ loadings. The colours indicate the group for which the variables have a maximal mean value. Variables with positive loadings (on the right of the Y axis) are correlated with each other and negatively correlated with variables with negative loadings (on the left of the Y axis).

Subsequently, a new sPLS-DA was performed using the Early and Established infection classes. Component 1 showed the greatest separation between the two groups (Fig. 3a) and identified the variables which contributed the most to the discrimination. High pFEV_1_, fast bacterial growth, high Abz hydrolysis, absence of *P. aeruginosa* isolated in the past 3 months, large green non-mucoid colonies, tobramycin susceptibility, and the presence of a LasR WT encoding gene were all characteristics that were correlated and overrepresented in the Early infection class. In contrast, older patients, longer time since first infection, *P. aeruginosa* infection within the last 3 months, mucoid and very small smooth white colonies, and monomicrobial infections were all characteristics correlated and overrepresented in the Established infection class (Fig. 3b). The cross-validation error obtained with this model was 3.2%, indicating considerably better performance than the initial sPLS-DA.

### Adaptation of *P. aeruginosa* versus co-infection

In order to assess the potential clonality of isolates collected from the same patient, Multilocus Sequencing Typing (MLST) was performed on a subset of isolates from the refined dataset, selecting those that were collected from the same patient and were in the same Leeds group (Supplementary Table S6). The isolates could be classified into two groups, termed Single Infection and Co-infection. In the Single Infection group, comprising 48% of the patients analysed, all isolates collected from a single patient shared the same sequence type (ST), yet exhibited genotypic (*lasB/lasR* sequence) or phenotypic (LasB production) differences. For certain patients (e.g. Patients 2, 10, 32, 75), this was the case even though the isolates were from the same sputum sample. In other patients (e.g. Patient 51), the same ST was maintained over time, across different sputum samples, although additional mutations appeared. These results support the occurrence of microevolution of *P. aeruginosa* to adapt to the CF lung environment. In the Co-infection group, comprising 52% of the patients analysed, isolates from the same patient presented different STs, indicating either the presence of multiple strains within the lungs at the same time (e.g., Patients 70 and 71), or sequential infections with different strains of *P. aeruginosa* (e.g., Patients 1, 16, 30). It is worth noting that patients with Intermittent or Chronic infections, were more abundant in the Co-infection group (91%), than in the Single infections group (70%).

## Discussion

This study evaluated 255 *P. aeruginosa* isolates collected from a cohort of 70 CF patients from the Toulouse region of France, over a period of 4.5 years. The patients were classified by the clinicians according to the Leeds criteria^25,26^, which are widely used to define the stage of *P. aeruginosa* infections in patients with CF. The *P. aeruginosa* isolated from the different stages of infection showed specific characteristics; for instance, *P. aeruginosa* from Chronic infections were more strongly associated with small colony phenotypes and showed a significantly higher prevalence of mucoidy, while showing a significantly lower prevalence of pigmentation, relative to the Intermittent and New infection isolates. These characteristics have already been described as typical of *P. aeruginosa* isolated from chronic infections^5,29,30^ and are associated with increased antibiotic resistance^5^ and reduced expression of virulence factors (such as LasB), relative to the WT PA01 strain^29^. The small colony phenotype observed here could correspond to the small colony variants (SCV) that have been previously described in the context of chronic lung infection in CF patients^31–33^. However, further characterization would be necessary to distinguish the SCV isolates from those that present a slow growth rate.

PCR amplification of the *lasB* locus confirmed the presence of this gene in all isolates, although alternative primers had to be used in some cases to achieve good amplification. Some studies have reported the *lasB* gene as absent in certain clinical strains^34–36^; however, another study that analysed 162 CF isolates also detected the *lasB* gene in all strains^37^. Moreover, an evaluation of the genomic data from a bronchiectasis *P. aeruginosa* collection published by Hilliam et al.^38^ confirmed that the *lasB* locus was also present in 100% of those isolates (data not shown). Finally, the analysis of the Pseudomonas.com global database revealed the presence of the *lasB* locus in 99.1% of the deposited genomes, independently of their origin. It is worth noting that those genomes lacking the *lasB* gene are mostly at the contig/scaffold assembly level, with only two at the chromosome/complete genome assembly level, suggesting that the *lasB* gene may be present within the gaps of the incomplete genomes, and only truly absent in a tiny minority of strains. Therefore, the discrepancies in the detection of *lasB*, between different PCR-based studies, may be due to the techniques used or the primers employed. The data presented provide strong evidence that the *lasB* gene is present in the vast majority of *P. aeruginosa* strains.

Analysis of the LasB amino acid sequences revealed nine variants, three of which accounted for 94.5% of the 255 CF isolates analysed. The same three major variants were identified in 87.3% of the Pseudomonas.com global database (n = 7890) and, interestingly, also in 83.1% of a collection of *P. aeruginosa* isolates from non-CF bronchiectasis patients (n = 189; data not shown)^38^. The similar distribution of LasB variants between the different databases suggests that this locus is well conserved among *P. aeruginosa* strains. Seven out of the nine LasB variants identified in this collection, including the three major variants, showed similar specific activities, with only the LasB-9 variant showing slightly lower specific activity^28^. These data indicate that most *P. aeruginosa*, regardless of their provenance, have the potential to express a functional LasB.

Consistent with the above observations, no significant differences in elastase activity were observed between isolates with different LasB variants. Interestingly, a small proportion of isolates (11.4%) were capable of producing LasB protein, as evidenced by Western blot, but showed no hydrolysis of the Abz substrate, despite having *lasB* sequences encoding active enzymes. Cigana et al*.*^22^ have previously shown that two strains isolated from the same patient 7 years apart, carried the same *lasB* sequence and produced similar amounts of protein by Western blot but had different levels of elastase activity. In that study, the first isolate showed a strong elastase activity, whereas the strain isolated during the chronic infection had no detectable elastase activity and presented pathoadaptive phenotypic traits. The authors suggested that the lack of elastase activity could be linked to incorrect processing of the protein. It is possible that a similar mechanism accounts for the lack of activity observed in certain strains in this study.

The expression of LasB is controlled by a complex QS network, responding to bacterial cell density and multiple environmental signals. Several QS transcriptional regulators have been identified in *P. aeruginosa*, however, the main regulator controlling the expression of *lasB* is the LasR activator^19^. Mutations leading to the inactivation of LasR have been described as an early adaptation to the CF lung environment and have been associated with the progression of lung disease in CF patients^39,40^. On the other hand, single-amino acid mutations in LasR have also been observed in *P. aeruginosa* isolates from chronic infections, some of which maintain their elastase activity^41^. Thus, we evaluated the occurrence of *lasR* variants in our collection of isolates, as well as the correlation of those variants with the hydrolysis of the Abz substrate. The *lasR* locus was successfully amplified and sequenced in 254 of the 255 isolates. Consistent with earlier studies, 63.8% of the strains presented a WT LasR, but a large number of variants were also identified, including 14.2% encoding a truncated LasR protein, which were associated with the lowest levels of LasB activity. Zupetic et al*.* similarly observed that mutant *lasR* genotypes were more abundant among low elastase *P. aeruginosa* strains isolated from respiratory infections in an ICU^42^. Moreover, we observed that the Chronic infection group (according to the Leeds Criteria) had the highest proportion of isolates carrying mutations in the *lasR* locus and the lowest level of LasB activity, supporting the importance of LasR in the expression of LasB. Unfortunately, due to the limited availability of multiple isolates from individual patients in this retrospective study, it was not possible to observe directly whether elastase activity was reduced in consecutive isolates over time (data not shown). To address this, prospective studies focused on obtaining comprehensive longitudinal data may be required.

To better understand the correlations between different variables (both bacterial and patient related) and their link to elastase activity, a supervised multivariate analysis was performed on a dataset that was refined so as to remove likely duplicate samples which might otherwise bias the analysis. This model did not discriminate between the three Leeds groups, most likely because Intermittent infections are poorly defined and their common characteristics difficult to determine^26^. This finding is in agreement with previous studies that have shown that many patients assigned to the Intermittent category are likely to have chronic infections^43,44^.

An MLST analysis of the strains from the refined dataset that were isolated from the same patient and, that were in the same Leeds group, identified clonally related isolates that had relevant genotypic and/or phenotypic differences, i.e., mutations in the *lasB*/*lasR* loci or differences in the activity of LasB. These results demonstrate that *P. aeruginosa* evolves and adapts to the CF lung environment and that several mutants originating from the same sequence type can coexist. Bragonzi et al*.*^30^ previously described the microevolution of *P. aeruginosa* in the CF context. In that study they showed that clonal isolates carried genomic rearrangements, mutations and variations in pathogenic islands, as well as phenotypic variations including mucoidy and virulence factors. Indeed, long-term *P. aeruginosa* infections have been characterized by the accumulation of mutations in diverse genes, including transcriptional regulators, virulence factors, antibiotic susceptibility and quorum sensing (*lasR*)^4^ Conversely, a group of patients presented multiple STs among their isolates, some of them isolated form a single sputum sample, and others isolated at different timepoints. Coinfections and sequential or intermittent infections by different strains of *P. aeruginosa* have also been described in the literature^45,46^ and seem to be a common phenomenon in the CF context.

For the purposes of this study, we sought to define a model that more accurately reflected the characteristics of the *P. aeruginosa* isolates and the clinical status of the patient. A hierarchical classification led to the identification of two distinct classes of isolates, which we termed Early and Established infection, based on their properties, that were then used in a second supervised multivariate analysis. In this case, the discrimination between the two groups was clear and the main discriminating variables were identified.

Our analysis revealed that characteristics such as lower LasB activity, mucoid phenotype, very small raised and smooth colonies and lack of colony pigmentation were overrepresented in the Established class. These traits were correlated with older patients, with lower pFEV_1_, that carried *P. aeruginosa* infections for a longer time, that have had *P. aeruginosa* infections in the last 3 months and had monomicrobial infections. These observations are consistent with reports that patients carrying lung-adapted, small colony variants of *P. aeruginosa* present poorer lung function and significantly lower pFEV_1_^33^.

In contrast, Early infection isolates presented higher LasB activity which correlated with other phenotypic traits, such as tobramycin and aztreonam susceptibility, fast bacterial growth, presence of pigmentation, medium or big flat non-mucoid colonies, as well as a WT LasR. These traits were associated with younger patients, carrying polymicrobial infections, with high pFEV_1_. In the context of the CF environment, “naïve” *P. aeruginosa* strains adapt through successive genetic modifications, including the overexpression of *algU*, mutations in *rpoN*, *gyrA* and other components of the DNA polymerase, that ultimately lead to a decrease in the growth rate, affecting antibiotic tolerance and persistence^47^. In our model, fast bacterial growth, reflecting higher growth rates, was overrepresented in the Early infection class. Moreover, this trait was correlated with greater susceptibility to tobramycin and aztreonam, which is consistent with the naïve phenotype.

It has previously been demonstrated that LasB plays an important role during *P. aeruginosa* infection and triggers host inflammation^48–50^. In turn, our data suggest that LasB is most important during the initial phase of the infection, since it is the Early infection class that is characterized by a higher LasB activity and the presence of a WT LasR. Mutations in the *lasR* locus leading to a loss of function have been associated with higher ICAM-1 response and increased neutrophilic pulmonary inflammation, increased tolerance to antibiotics, as well as a reduced elastolytic and caseinolytic activities and reduced production of pyocyanin^51,52^. In our study, loss of elastase activity during long-term (chronic) infection appears to be correlated to the loss of LasR function, rather than any mutation in the *lasB* locus itself, although LasR does not seem to be solely responsible for this phenotype. Indeed, 25.3% of isolates which showed no LasB activity (i.e. negative for hydrolysis of Abz) exhibited a WT *lasR* genotype, thus other changes in the regulation or processing of LasB are required to explain this phenotype. In agreement with this, while the presence of a WT LasR was clearly associated with the Early infection class, mutations in *lasR* were not identified by the sPLS-DA as a critical variable to characterize the Established infection.

Our study combined clinical and experimental data associated with 255 *P. aeruginosa* isolates from a single CF regional centre. The results provide a ‘big picture’ of strain diversity and evolutionary development, confirming many previous observations made with more limited datasets, but also revealing the huge complexity of *P. aeruginosa* phenotypic and genomic diversity within this population and within individual patients. Multivariate analysis was performed to identify variables that differentiate between Early and Established infection *P. aeruginosa* isolates, and specifically those phenotypic and genotypic traits that are correlated to the expression of active LasB. In conclusion, our data indicate that *P. aeruginosa* isolates from CF patients express higher levels of LasB elastase activity during the early stages of infection and that once a chronic infection is established the elastase activity is significantly diminished in many strains, coincident with the emergence of mutations in *lasR*, reduced growth rate and the appearance of antibiotic resistance. These findings suggest that, in CF patients, anti-virulence therapies targeting the LasB elastase may be most effective for the treatment of early-stage infections, prior to the establishment of chronicity.

## Materials and methods

### Patients and isolates

This study was designed in accordance with the World Medical Association Declaration of Helsinki and conducted in compliance with the French legislation applicable to non-interventional retrospective studies (MR-004). According to the French ethic and regulatory law, studies based on the exploitation of usual care data and samples did not have to be submitted to a specific ethic committee but had to be declared or covered by reference methodology of the French National Commission for Informatics and Liberties (CNIL). The patients were informed that their codified data will be used for the study and their informed consent (non-opposition) was collected and a trace was kept in each medical file. Antabio SAS signed a commitment of compliance to the reference methodology MR-004 of the CNIL (CNIL number: 2229447 v 0). The study was thus approved by Antabio SAS after evaluation by the data protection officer and confirmation that the ethical requirements were respected. This study was registered under the number: PEi-042.

255 *P. aeruginosa* were isolated from sputum samples obtained from 70 CF patients from several CF centres in the Toulouse region of France. Samples were collected during routine visits (every 3 months) and episodes of pulmonary exacerbation. Sputum samples were collected from CF patients according to the French Guidelines for cystic fibrosis^53^.

### Isolation of *P. aeruginosa* from sputum samples

Sputum samples were homogenized with an equal volume of dithiothreitol for 30 min at 37 °C. For *P. aeruginosa* isolation, 10 µL samples of homogenate were plated onto Cetrimide agar plates (bioMérieux, Marcy l’Etoile, France) and incubated at 35(± 2) °C for 48–120 h. A single colony representing each morphotype was isolated and analysed using MALDI-TOF mass spectrometry (MALDI BioTyper, Bruker) to confirm *P. aeruginosa* identity. Antimicrobial susceptibility testing (AST) was performed using standard disk diffusion methodology according to CA-SFM/European Committee on Antimicrobial Susceptibility Testing (EUCAST) Guidelines^54^. *P. aeruginosa* ATCC 27853 strain was used as control. AST was conducted with the following antibiotics: aztreonam (ATM), tobramycin (TOB), ticarcillin (TIC), ticarcillin-clavulanic acid (TIM), piperacillin (PIP), piperacillin-tazobactam (TZP), ceftazidime (CAZ), cefepime (FEP), imipenem (IMP), meropenem (MEM), gentamycin (GEN), amikacin (AMK), levofloxacin (LVX), ciprofloxacin (CIP), fosfomycin (FOF) and colistin (CST). Only the AST results for ATM and TOB were considered for statistical analysis, since these antibiotics are the standard of care for *P. aeruginosa* infections in CF. Visual determination of mucoid phenotype was also evaluated on a subset of 137 isolates immediately after the isolation to obtain a faithful reflection of the phenotype of these strains in the lungs, and was not re-evaluated thereafter to avoid the emergence of revertants. Strains were stored at − 20 °C in Brain Heart Infusion (BHI) broth containing 10% glycerol*.*

### Genotypic and phenotypic characterization

Genomic DNA was extracted from each isolate for PCR amplification of the *lasB* and *lasR* loci. First, approximately 4 × 10^8^ bacterial cells from an exponentially growing culture were harvested by centrifugation and the pellet was resuspended in TE buffer (Tris–HCl pH 8 100 mM, EDTA pH 8 10 mM). The cells were frozen at − 80 °C for 20 min and then transferred to a screw cap tube with glass beads. The samples were homogenized in the FastPrep-24 5G (MP Biomedicals) (5 cycles of 30 s at speed 5.5), centrifuged and the supernatant containing the genomic DNA was recovered. An ethanol precipitation was performed to concentrate the DNA.

PCR amplification of *lasB* was first performed with primers AmpF1/AmpR1 (Supplementary Table S5), and a second pair (LasB_EF/LasB_ER) used if the first set failed. *lasR* amplification was performed with the lasR_EF/lasR_ER primers (Supplementary Table S5). PCR products were purified using the PureLink™ Pro 96 PCR Purification Kit (Invitrogen) and sequenced by Macrogen Europe, using specific primers.

A total of 98 *P. aeruginosa* isolates from the refined dataset were examined for colony morphology (shape, size, margin, elevation) and pigmentation after overnight growth on LB agar at 37 °C. Colony pigmentation was classified as white (no pigmentation), green or red.

### LasB activity and expression in *P. aeruginosa* supernatants

Flasks containing LB broth were inoculated at an OD_600_ = 0.015 with an overnight *P. aeruginosa* culture and incubated for 24 h at 37 °C at 180 rpm. Cultures were centrifuged for 10 min at 5000 rpm, and supernatants were filtered (0.22 µm pore) and stored at − 80 °C. For each experiment, the PAO1 strain and a Δ*lasB* derivative were used as positive and negative controls, respectively.

Hydrolysis of the fluorogenic peptide substrate 2-aminobenzoyl-Ala-Gly-Leu-Ala-4-nitrobenzylamide (Abz; Enzo Life Sciences) was used to assess LasB activity on culture supernatants. The assay was performed in 50 mM Tris–HCl (pH 7.4) supplemented with 2.5 mM CaCl_2_ and followed using an Envision microplate reader (Perkin Elmer) at 315 nm excitation and 430 nm emission wavelengths. The results were expressed in RFU/mL of supernatant. Supernatants that showed less than a baseline threshold of 1.88 × 10^8^ RFU/mL (calculated as the mean of the negative controls plus three times the SD), were considered negative. All isolates were tested once and if the results were below the threshold for positivity two further replicates were performed to confirm the negative result.

The presence of LasB protein in the supernatants was evaluated by Western blot analysis. Twenty-five microlitres of culture supernatants were mixed with an equal volume of a Laemmli blue 2 × buffer and denatured by heating at 95 °C for 5 min. Ten microlitres of protein sample were run on a 12% Mini-Protean TGX Precast Protein Gels (Bio-Rad). After electrophoresis, proteins were transferred to a 0.2 µm PVDF membrane. After 1 h in TBST (Tris buffered saline-Tween 20) 5% skim milk to saturate the non-specific sites, membranes were then blotted 1 h at RT in TBST with 0.5% skim milk in the presence of the LasB primary antibody (provided by Efrat Kessler’s lab, Tel Aviv University, Israel). Primary antibody was detected using secondary antibody conjugated with alkaline phosphatase and revealed with the NBT/BCIP substrate (Sigma).

### Multilocus sequencing typing

MLST was performed as described previously^55^. Briefly, the seven genes, *ascA*, *aroE*, *guaA*, *mutL*, *nuoD*, *ppsA* and *trpE*, were amplified by PCR using the primers designed by Curran et al.^55^. The amplifications were performed using the GoTaq G2 Hot Start Green Master Mix (Promega), the forward and reverse primers at a final concentration of 0.4 μM, DMSO 6% and 100 ng of gDNA (4 ng/μL) in a final volume of 25 µL. The amplification program was set as follows: initial denaturation at 95 °C for 5 min followed by 30 cycles of denaturation at 95 °C for 45 s, hybridization at 55 °C for 1 min and elongation at 72 °C for 1 min, followed by a final elongation at 72 °C for 7 min. After amplification, 5 μL of PCR products were run on a 1.3% agarose gel to verify the size of the amplicons. Samples were then stored at − 20 °C before being sent to Microsynth for purification and sequencing. Reconstructed gene sequences were analysed to determine their allelic profile. Finally, for each strain, the set of allelic profiles obtained was used to identify the ST profile^56^.

### Statistical analysis

All clinical data supplied by the Toulouse CF centres, and the experimental data were combined into a single dataset. Descriptive measures for numerical variables (means, medians, SD and ranges) were calculated using Vortex (Dotmatics).

The following analyses were all conducted in the software R^57^. Comparisons of the expected distribution of a phenotypic trait and the actual distribution within the New, Intermittent and Chronic infection groups of isolates were done using the Pearson’s Chi-squared test. The test was run using the ‘chisq.test’ function from the ‘stats’ package^57^.

A refined dataset was created by eliminating likely duplicate isolates in order to minimise bias during the statistical analysis. The elimination criteria are detailed in the workflow in Supplementary Fig. S4. Isolates that were classified as ‘New infection’ for patients older than 30 years-old and isolates for which the clinical data were incomplete were also excluded. All the subsequent statistical analysis were performed with the refined dataset.

Boxplots were generated using the ‘ggboxplot’ function from the ‘ggpubr’ package^58^. To determine if there were significant differences between the medians of the different groups, a Kruskal–Wallis test was performed using the ‘kruskal.test’ function. The pairwise comparison on the medians was performed with the Dunn’s test, using the ‘dunnTest’ function from the ‘FSA’ package^59^.

The refined dataset was normalized for further analysis. The numerical variables were centred and scaled, and the categorical variables were transformed into dummy variables using the ‘fastDummies’ package^60^. The classification of the isolates was performed using a clustering analysis with the ‘hclust’ function. Sparse partial least squares discriminant analysis (sPLS-DA) was performed using the ‘splsda’ function of the ‘mixOmixs’ package^61^. The optimal number of components in the model was defined with the ‘perf’ function using three-fold cross-validation repeated 1000 times. The number of selected variables in the model was defined with the function ‘tune.splsda’.

### Supplementary Information

Supplementary Information 1.

Supplementary Information 2.

## Data Availability

All data generated or analysed during this study are included in this published article (and its Supplementary Information files).
