# Supplementary material for: Higher levels of Pseudomonas aeruginosa LasB elastase expression are associated with early-stage infection in cystic fibrosis patients
Source: Sci Rep. 2023 Aug 30;13:14208. doi: 10.1038/s41598-023-41333-9 (PMC10468528; doi:10.1038/s41598-023-41333-9)

Supplementary data

Title

Higher levels of *Pseudomonas aeruginosa* LasB elastase expression are associated with early-stage infection in Cystic Fibrosis patients

Authors

Agustina Llanos*[1], Pauline Achard[1], Justine Bousquet[1], Clarisse Lozano[1], Magdalena Zalacain[1], Carole Sable[1], Hélène Revillet[2,3], Marlène Murris[4], Marie Mittaine[5], Marc Lemonnier[1], Martin Everett[1]

[1] Antabio SAS, Labège, France

[2] Service de Bactériologie-Hygiène, CHU de Toulouse, Toulouse, France

[3] IRSD, INSERM, Université de Toulouse, INRAE, ENVT, UPS, Toulouse, France

[4] Adult Cystic Fibrosis Centre, Pulmonology Unit, Hôpital Larrey, CHU de Toulouse

[5] Hôpitaux de Toulouse, Toulouse, France

Tables

Table S1. Comparison of the observed prevalence of a phenotypic trait within a group of isolates and the expected prevalence if that trait was randomly distributed among the entire population. The Chi2 test was used and the p-values were adjusted using the Holm method.

Small colony phenotype							
	Observed	%	Expected	%	p-value	Adjusted p-value	Significance
Chronic	15/32	46.88	21/98	21.40	4.42×10^{-4}	1.33×10^{-3}	**
Intermittent	4/25	16.00	21/98	21.40	0.404	0.404	ns
New	2/41	4.88	21/98	21.40	9.89×10^{-3}	1.98×10^{-2}	*
**Pigmentation**							
	Observed	%	Expected	%	p-value	Adjusted p-value	Significance
Chronic	10/32	31.25	54/98	55.10	6.68×10^{-3}	0.020	*
Intermittent	17/25	68.00	54/98	55.10	0.195	0.332	ns
New	27/41	65.85	54/98	55.10	0.166	0.332	ns
**Mucoid**							
	Observed	%	Expected	%	p-value	Adjusted p-value	Significance
Chronic	27/58	46.55	30/137	21.90	5.64×10^{-6}	1.69×10^{-5}	****
Intermittent	0/39	0.00	30/137	21.90	9.43×10^{-4}	1.89×10^{-3}	**
New	3/40	7.32	30/137	21.90	0.028	0.028	*

Table S2. Prevalence of the different LasR variants in the isolate collection from this study. The *lasR* gene was amplified and sequenced from 254 out of the 255 isolates included in this study. The LasR sequence from the *P. aeruginosa* PAO1 strain was considered the reference sequence (WT).

Type of mutation	AA mutations	This study (n=254)	
		Number of strains	Prevalence in the population (%)
Reference sequence (PAO1)	WT	162	63.8
Amino-acid substitution or Small deletion	A21T	6	22.0
	A50G	1	
	N55K	14	
	R61C	1	
	R61S	1	
	H78R	4	
	G162C	1	
	T178I	3	
	E183K	1	
	V184A	4	
	C188Y	3	
	G191D	1	
	E196D	1	
	V199A	2	
	V208A	1	
	R217W	1	
	K218N	1	
	V221E	5	
	R244C	1	
	A231V	2	
	T222-S223del	1	
	S77-Q81del	1	
Nonsense mutation	Q45X	3	7.9
	Q81X	4	
	E133X	10	
	W195X	2	
	S204X	1	
Frameshift mutation	A108fsX111	1	6.3
	K155fsX158	1	
	Q24fsX114	1	
	L36fsX114	1	
	V72fsX115	1	
	L114fsX233	6	
	G120fsX236	2	
	E139fsX222	2	
	E139fsX211	1	

Table S3. Kruskal-Wallis and Dunn's tests results for the comparisons of Abz hydrolysis per mL of SN among the different groups: the LasB variants, LasR variants and the Leeds' stages of infection. *p*-value: ** <0.01; *** <0.001; **** <0.0001

	**Test**	**p-value**	
ABZ hydrolysis per mL of SN between LasB variants	Kruskal-Wallis	0.336 (ns)	
	Dunn's (adjusted p-value)	LasB WT - LasB-2	0.89
		LasB WT - LasB-3	0.53
		LasB WT - LasB variants	1
		LasB-2 - LasB-3	0.62
		LasB-2 - LasB variants	1
		LasB-3 – LasB variants	1
ABZ hydrolysis per mL of SN between LasR variants	Kruskal-Wallis	1.24e-6 (****)	
	Dunn's (adjusted p-value)	LasR WT - LasR variants	3.44×10^{-4} (***)
		LasR WT - LasR truncated	3.92×10^{-5} (****)
		LasR variants - LasR truncated	0.25
ABZ hydrolysis per mL of SN between Leeds ' classes	Kruskal-Wallis	0.009 (**)	
	Dunn's (adjusted p-value)	New - Intermittent	0.39
		New - Chronic	7.03×10^{-3} (**)
		Chronic - Intermittent	0.11

Table S4. Comparison of different variables among isolates from the Early and the Established infection classes.

		Early infection group	Established infection group
Number of isolates (n=102)		72	30
Patient characteristics†	Age, median (range)	11 (1-30)	28 (15-46)
	pFEV1, median (range)	87 (30-125)	67 (30-111)
Stage of infection (Leeds criteria), number of isolates (%)	New isolates	42 (58.3%)	0 (0%)
	Intermittent isolates	20 (27.8%)	7 (23.3%)
	Chronic isolates	10 (13.9%)	23 (76.7%)
LasB type, number of isolates (%)	LasB-1 (WT)	24 (33.3%)	14 (46.7%)
	LasB-2	22 (30.6%)	6 (20.0%)
	LasB-3	19 (26.4%)	7 (23.3%)
	LasB-4 to LasB-9	7 (9.7%)	3 (10.0%)
LasR type, number of isolates (%)	LasR WT	49 (68.1%)	15 (50.0%)
	LasR variants	15 (20.8%)	9 (30.0%)
	LasR truncated	8 (11.1%)	6 (20.0%)
ABZ hydrolysis in RUF per mL of SN, median (range)		1.5×10^{11} (1.31×10^{8}-4.23×10^{11})	1.56×10^{8} ($1.26\times10^{+8}$-3.82×10^{11})
Tobramycin resistance, number of isolates	R	2 (2.8%)	6 (20.0%)
	S	65 (90.3%)	13 (43.3%)
	N/A	5 (6.9%)	11 (36.7%)

Table S5. List of primers used in this study.

Primer	Primer sequence (5' – 3')	Amplicon size (bp)	Purpose
AmpF1	GCGAAATCAAGGCTACCTG	1833	External primers for *lasB* amplification and sequencing
AmpR1	CTGAACTTTAGACCGGGTTC		
lasB_EF	TACACGAAAGCACCGTCGAA	1954	External primers for *lasB* amplification and sequencing
lasB_ER	GGTCGGTCTTGGAATAAGTGC		
lasR_EF	CAACTCTATAGAGTGGGCTGAC	1021	External primers for *lasR* amplification and sequencing
lasR_ER	CTTCGGGATAAGCCAATCCT		

Table S6. STs found in a selection of isolates from the refined dataset.

Patient ID	Strain number	Stage of Infection	Time (months) since first infection	LasB variant	LasR variant	Abz hydrolysis	ST
**Single infections**							
10	NTBC469.2	New	0	LasB_1	LasR_WT	Positive	363
	NTBC469.1	New	0	LasB_2	LasR_WT	Positive	363
66	NTBC653	New		LasB_3	LasR_trunc	Negative	313
	NTBC652	New		LasB_3	LasR_trunc	Positive	313
75	NTBC631	New	0	LasB_2	LasR_var	Negative	244
	NTBC630.1	New	0	LasB_2	LasR_var	Positive	244
4	NTBC655	Inter	183	LasB_2	LasR_WT	Positive	633
	NTBC489.1	Inter	206	LasB_2	LasR_WT	Negative	633
27	NTBC485	Inter	194	LasB_1	LasR_WT	Negative	865
	NTBC466.1	Inter	203	LasB_1	LasR_WT	Positive	865
31	NTBC636	Inter	235	LasB_1	LasR_WT	Positive	4070
	NTBC495	Inter	249	LasB_2	LasR_WT	Positive	4070
51	NTBC512.2	Inter	200	LasB_1	LasR_WT	Positive	446
	NTBC572	Inter	240	LasB_3	LasR_WT	Negative	446
	NTBC535	Chronic	248	LasB_3	LasR_WT	Negative	446
	NTBC601	Chronic	253	LasB_3	LasR_WT	Positive	446
2	NTBC617	Chronic	245	LasB_2	LasR_trunc	Negative	676
	NTBC616	Chronic	245	LasB_2	LasR_WT	Positive	676
32	NTBC599	Chronic	360	LasB_1	LasR_var	Negative	2498
	NTBC598.1	Chronic	360	LasB_1	LasR_WT	Positive	2498
46	NTBC606.1	Chronic		LasB_1	LasR_WT	Negative	3243
	NTBC605	Chronic		LasB_1	LasR_var	Positive	3243
**Co-infections**							
1	NTBC501	New	0	LasB_1	LasR_WT	Positive	395
	NTBC448	New	27	LasB_3	LasR_WT	Negative	253

Patient ID	Strain number	Stage of Infection	Time (months) since first infection	LasB variant	LasR variant	Abz hydrolysis	ST
16	NTBC479	Inter	256	LasB_var	LasR_var	Negative	260
	NTBC531	Inter	285	LasB_1	LasR_trunc	Negative	17
30	NTBC442	Inter	33	LasB_1	LasR_WT	Positive	274
	NTBC590	Inter	49	LasB_3	LasR_var	Positive	1158
39	NTBC582	Inter		LasB_var	LasR_var	Positive	3218
	NTBC483	Inter		LasB_3	LasR_WT	Positive	1632
47	NTBC449	Inter	8	LasB_3	LasR_WT	Positive	1567
	NTBC566	Inter	14	LasB_3	LasR_var	Positive	1567
	NTBC557	Inter	16	LasB_1	LasR_var	Negative	2099
54	NTBC453	Inter		LasB_3	LasR_WT	Positive	298
	NTBC563	Inter		LasB_2	LasR_WT	Positive	285
62	NTBC487	Inter	1	LasB_2	LasR_var	Negative	1818
	NTBC488.1	Inter	1	LasB_2	LasR_var	Positive	244
	NTBC518	Inter	36	LasB_3	LasR_WT	Positive	235
23	NTBC664	Chronic	181	LasB_1	LasR_var	Positive	2099
	NTBC556	Chronic	217	LasB_3	LasR_WT	Positive	1567
	NTBC538	Chronic	221	LasB_1	LasR_var	Negative	2099
48	NTBC658	Chronic		LasB_1	LasR_trunc	Positive	480
	NTBC537	Chronic		LasB_2	LasR_WT	Positive	244
	NTBC457	Chronic		LasB_1	LasR_WT	Positive	480
	NTBC584.1	Chronic		LasB_3	LasR_WT	Positive	253
70	NTBC620.2	Chronic	294	LasB_1	LasR_trunc	Negative	1026
	NTBC620.1	Chronic	294	LasB_var	LasR_var	Negative	251
	NTBC622	Chronic	294	LasB_1	LasR_var	Positive	969
	NTBC619.2	Chronic	294	LasB_3	LasR_WT	Positive	446
	NTBC619.1	Chronic	294	LasB_1	LasR_WT	Positive	969
71	NTBC626	Chronic	206	LasB_3	LasR_var	Negative	279

Patient ID	Strain number	Stage of Infection	Time (months) since first infection	LasB variant	LasR variant	Abz hydrolysis	ST
	NTBC628	Chronic	206	LasB_var	LasR_var	Negative	1756
	NTBC627	Chronic	206	LasB_var	LasR_WT	Positive	1756
67	NTBC470*	New	0	LasB_2	LasR_WT	Negative	
	NTBC629	New	56	LasB_3	LasR_trunc	Negative	446

*The strain could not be recovered from the glycerol stock.

Figures

Figure S1. Scatter plot representing the sputum samples obtained over time from the 70 patients and from which *P. aeruginosa* was isolated. In some cases, more than one *P. aeruginosa* isolate was obtained from a single sputum sample. The patients' ID is represented in the Y axis, versus the date of sampling in the X axis. Each patient is represented in a different colour. Samples from the same patient are linked by a dashed line.

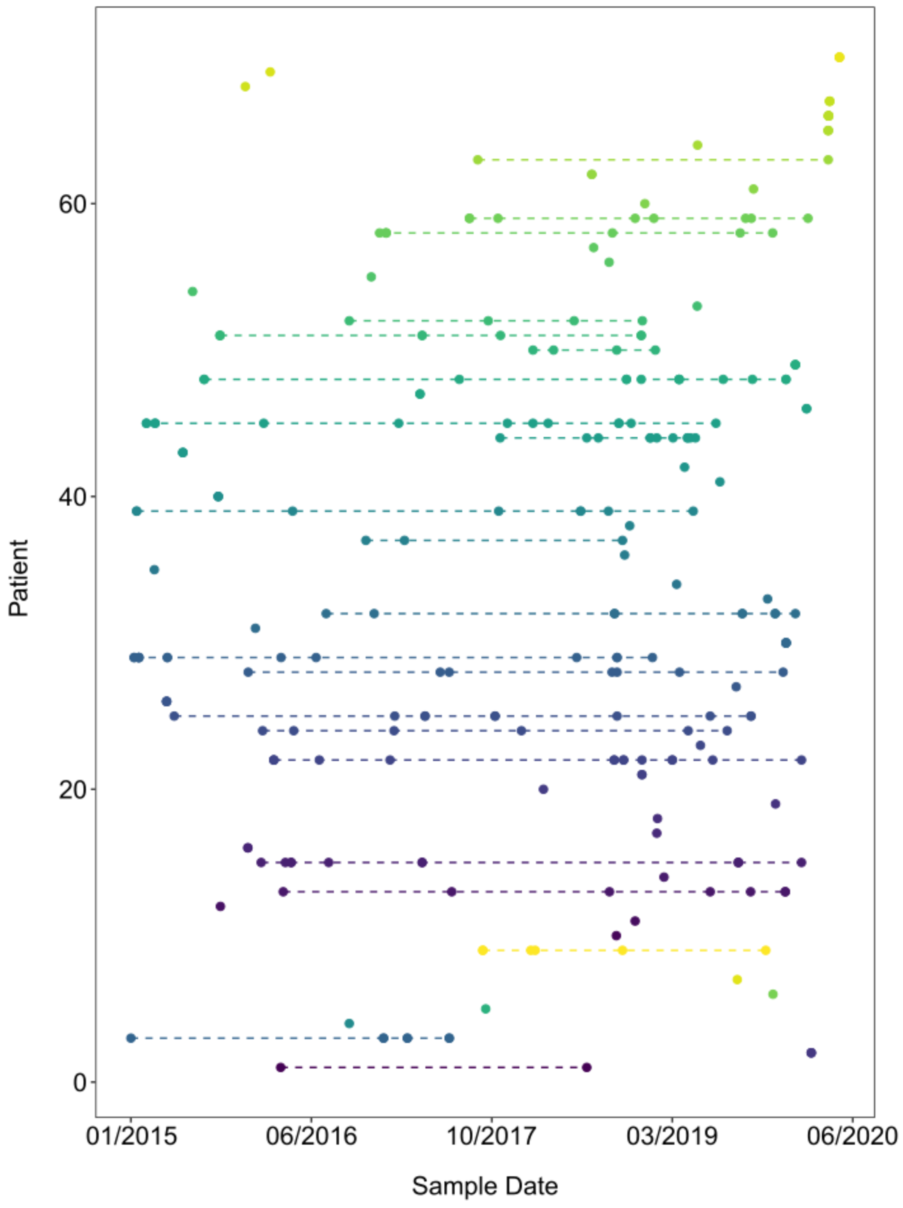

Figure S2. Comparison of the observed prevalence (bars) of a phenotypic trait within a group of isolates and the expected prevalence (black lines) if that trait was randomly distributed among the entire population. The Chi2 test was used and the p-values were adjusted using the Holm method. Significant differences between the observed and the expected prevalence are indicated by the stars on top of the bars. *p*-value: ns>0.05, *<0.05; ** <0.01; *** <0.001; **** <0.0001.

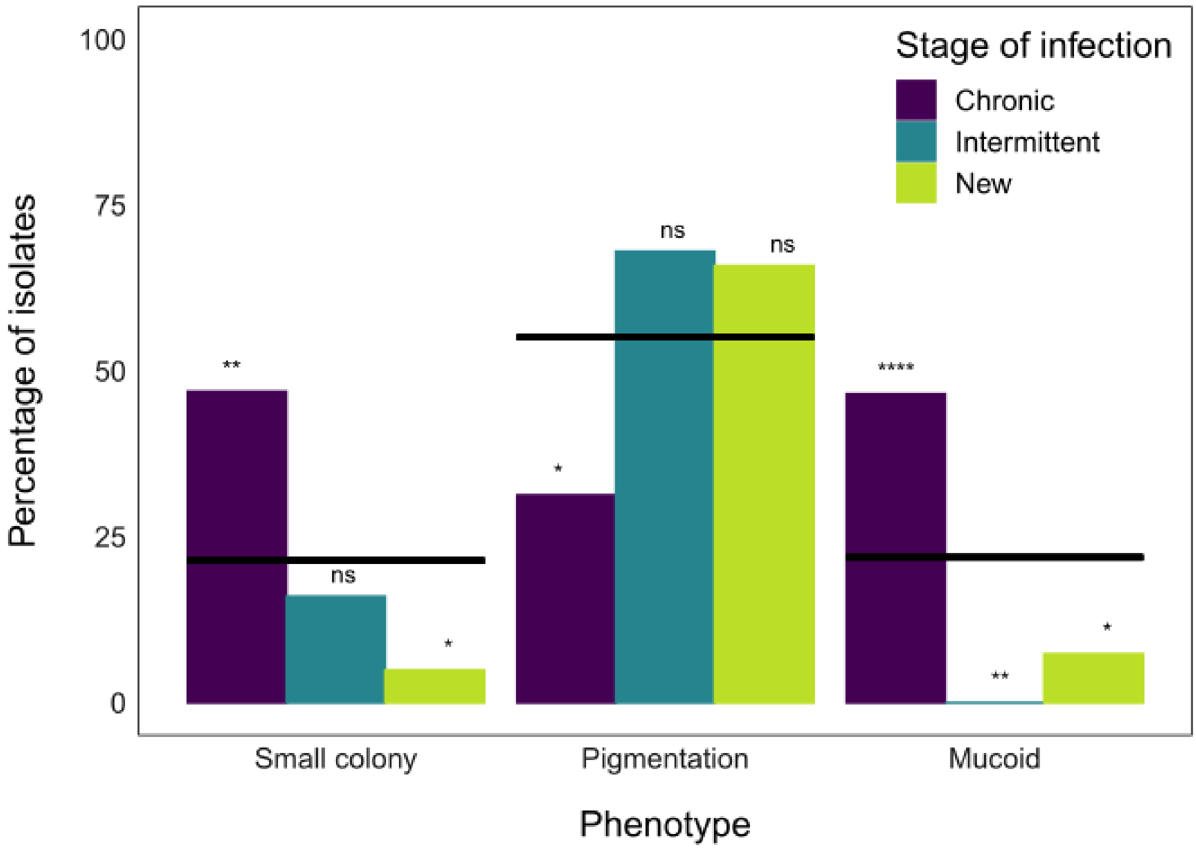

Figure S3. Detection of LasB in *P. aeruginosa* supernatants by Western blot. The Abz hydrolysis (positive or negative), LasB variant number and LasR variant (t: truncated, WT: wild type) corresponding to each sample are indicated below each lane. No modifications were done to the blot image.

M 1 2 3 4 5 6 7 8 9 10

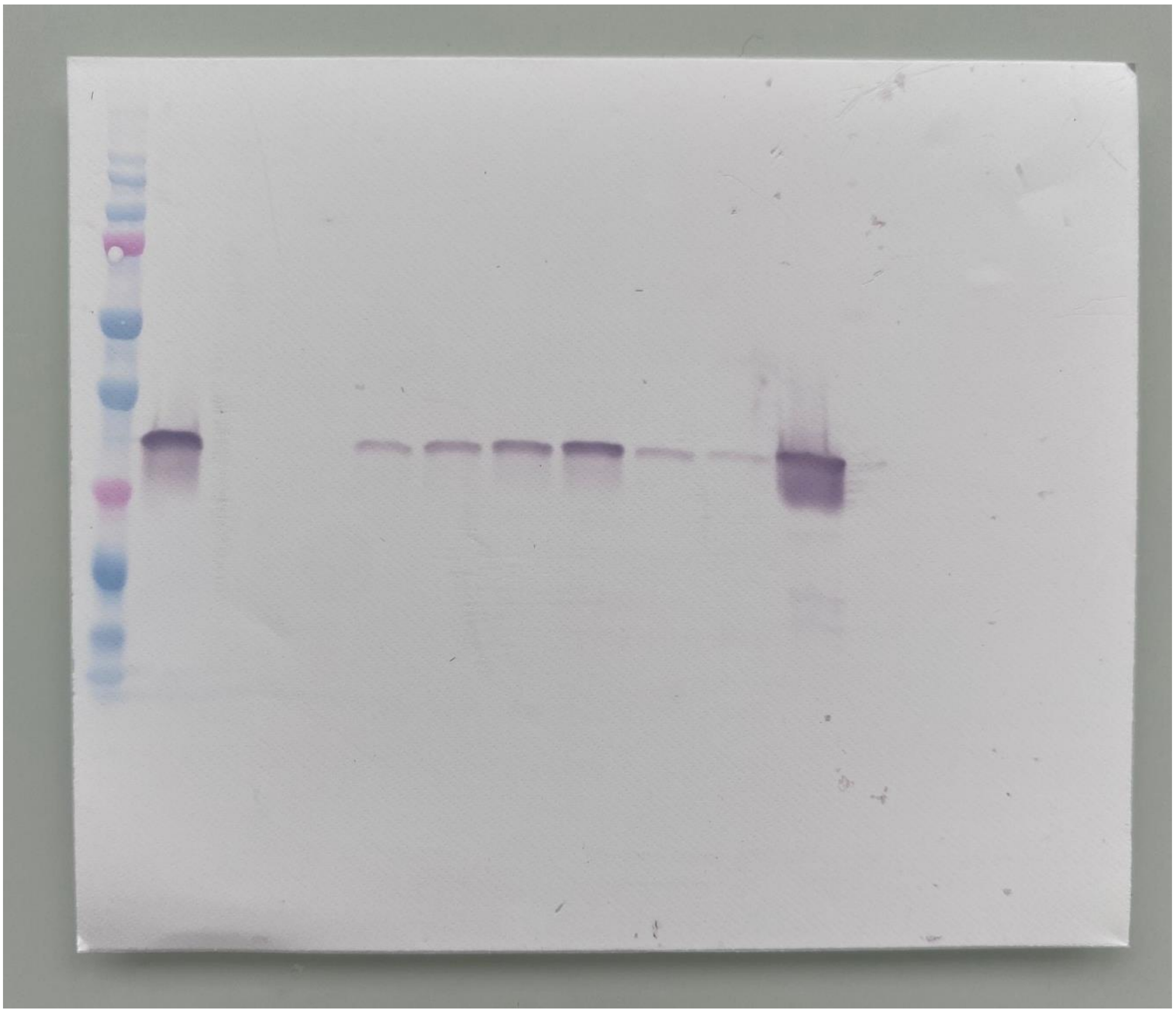

**Abz**	+	-	-	+	+	+	+	-	-	+
**LasB**	1	2	2	1	1	1	1	3	3	1
**LasR**	--	t	t	WT	WT	WT	WT	WT	WT	WT

M: Molecular weight marker
1: Purified LasB protein
2: NTBC444 n=1
3: NTBC444 n=2
4: NTBC524.2 n=1
5: NTBC524.2 n=2
6: NTBC569 n=1
7: NTBC569 n=2
8: NTBC572 n=1
9: NTBC572 n=2
10: PAO1

Figure S4. Workflow describing the selection process for the refined dataset, which resulted in 102 isolates. The LasB activity status was defined according to the Abz hydrolysis results. Abz $>1.88\times10^8$ RFU/mL of supernatant, positive LasB activity and Abz $\leq1.88\times10^8$ RFU/mL of supernatant, negative LasB activity.

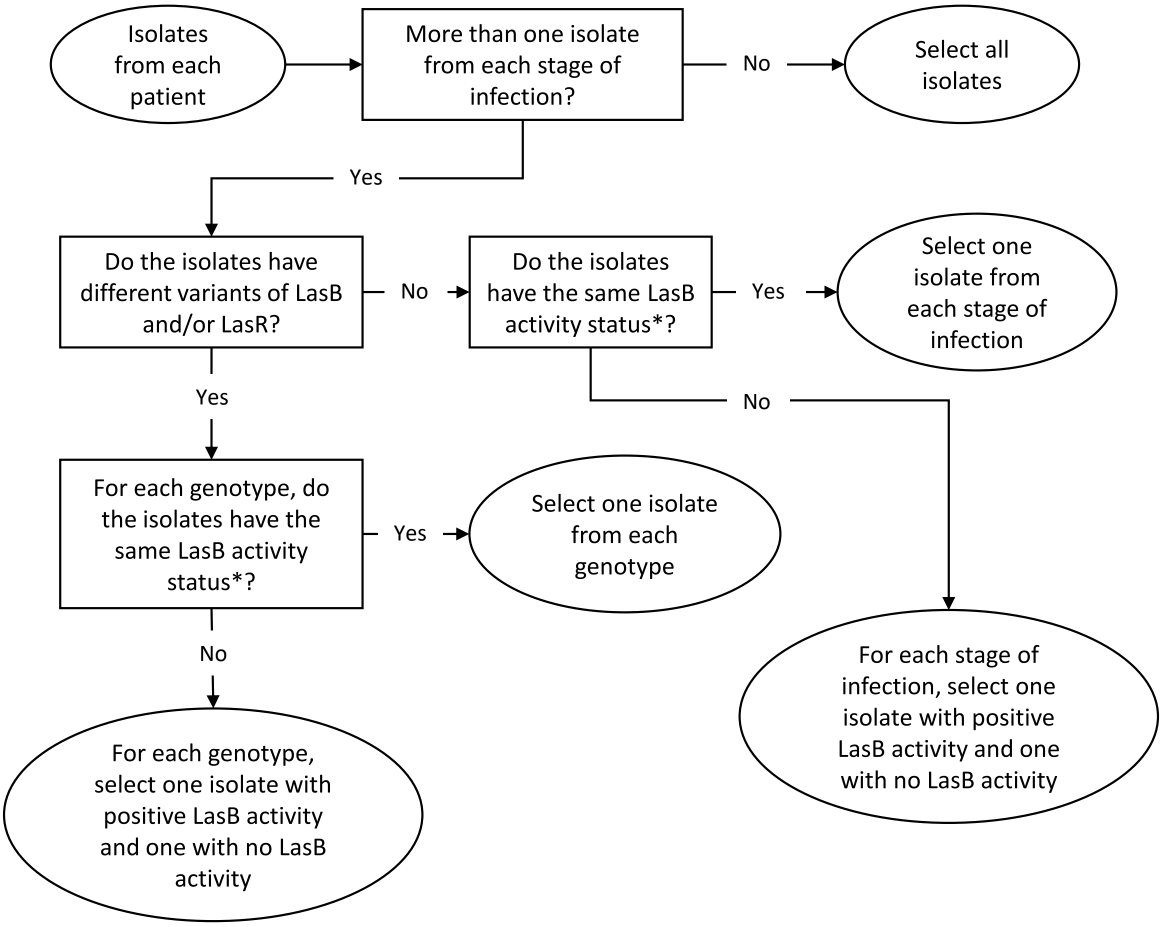

Figure S5. Individual plots of the sPLS-DA using the Leeds' New, Intermittent and Chronic infection groups.

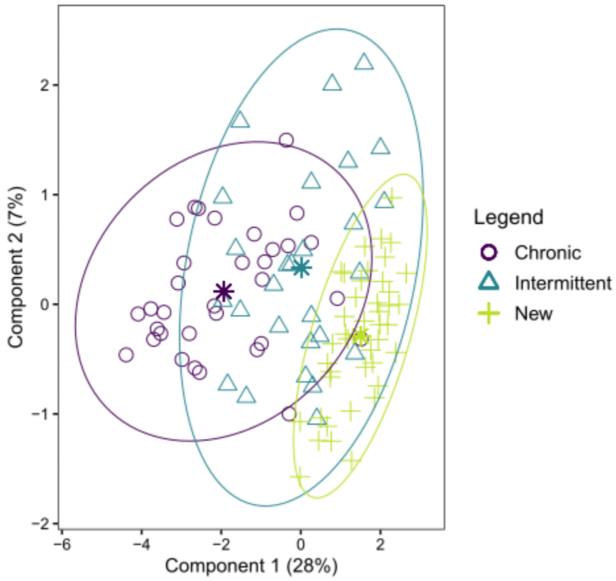

Supplement: Supplementary file 1 — Supplementary Information 1. [file 41598_2023_41333_MOESM1_ESM.pdf]
